# Lack of Endogenous IL-10 Enhances Production of Proinflammatory Cytokines and Leads to *Brucella abortus* Clearance in Mice

**DOI:** 10.1371/journal.pone.0074729

**Published:** 2013-09-17

**Authors:** Patrícia P. Corsetti, Leonardo A. de Almeida, Natália B. Carvalho, Vasco Azevedo, Teane M. A. Silva, Henrique C. Teixeira, Ana C. Faria, Sergio C. Oliveira

**Affiliations:** 1 Departmento de Bioquimica e Imunologia, Instituto de Ciencias Biologicas, Universidade Federal de Minas Gerais, Belo Horizonte-Minas Gerais, Brazil; 2 Departmento de Biologia Geral, Instituto de Ciencias Biologicas, Universidade Federal de Minas Gerais, Belo Horizonte-Minas Gerais, Brazil; 3 Departmento de Clinica e Cirurgia Veterinária, Escola de Veterinária, Universidade Federal de Minas Gerais, Belo Horizonte-Minas Gerais, Brazil; 4 Departmento de Microbiologia, Imunologia e Parasitologia, Universidade Federal de Juiz de Fora, Juiz de Fora-Minas Gerais, Brazil; University of São Paulo, Brazil

## Abstract

IL-10 is a cytokine that regulates the balance between pathogen clearance and immunopathology. *Brucella abortus* is an intracellular bacterium that causes chronic disease in humans and domestic animals. Here we evaluated the contribution of IL-10 in host immune response and pathology during *B. abortus* infection. To assess the role of IL-10 *in vivo*, IL-10 knockout (KO) or 129 Sv/Ev (wild-type) mice were infected with *B. abortus* and the number of viable bacteria from the spleen was determined at 1, 2, 3, 6 and 14-weeks postinfection. IL-10 KO mice showed reduced bacterial loads in the spleen when compared to wild-type mice during all time points studied. Additionally, at 14-weeks postinfection IL-10 KO mice had totally cleared the infection. This clearance was preceded by an enhanced IFN-γ, TNF-α and IL-17 responses in both the serum and the spleen of IL-10 KO mice. Additionally, dendritic cells from infected IL-10 KO mice produced elevated levels of IL-12 and TNF-α compared to wild-type animals. Histopathology analysis was performed and both KO and wild-type mice developed multifocal granulomas and necrosis in the liver. However, at six-weeks postinfection reduced numbers of granulomas was detected in IL-10 KO mice compared to wild-type animals. This reduced liver pathology at later stage of infection was accompanied by increased numbers of CD4+CD25+foxp3+ T cells and expression of TGF-*β* in IL-10 KO splenocytes. Taken together, our findings demonstrate that IL-10 modulates the proinflammatory immune response to *B. abortus* infection and the lack of IL-10 increases resistance to *Brucella* infection.

## Introduction

In general bacterial infections induced by Gram-negative microorganisms are associated with an acute inflammatory reaction, which represents the principal local defense against spread of the infection [1]. Many of the severe complications of bacterial infections result from excessive immune activation [2]. To increase the immune-mediated clearance of pathogens and infected host cells it is demonstrated that the maximal pathogen control does not necessarily lead to minimal disease [3], [4], [5], highlighting the essential role for immunoregulatory components of the immune response in limiting pathology. Among the immunoregulatory components related to prevent the exacerbated proinflammatory response against Gram-negative bacteria, IL-10 is considered a central immunoregulator antagonizing the excessive Th1 and CD8+ T cell responses [2]. IL-10 inhibits proinflammatory responses from innate and adaptive immunity, and it prevents the lesions in tissues caused by exacerbated adaptive immune responses [6].

*Brucella abortus* is a Gram-negative, facultative intracellular coccobacillus which causes brucellosis, a chronic inflammatory disease, in humans and in cattle. In humans *B. abortus* causes undulant fever, endocarditis, arthritis and osteomyelitis and, in animals, it leads to abortion and infertility resulting in serious economic losses [7], [8]. The protective response against *B. abortus* infection requires CD4^+^ and CD8^+^ T lymphocytes, Th1-type cytokines such as interferon-gamma (IFN-γ) and tumor necrosis factor (TNF-α), and activated macrophages and dendritic cells [9], [10]. Additionally, IFN-γ was demonstrated to be a critical cytokine for host control of *Brucella* infection [9], [11]. IL-10 is known to affect production of Th1 cytokines, including IFN-γ, by acting on the antigen-presenting cells capacity of the macrophages inhibiting MHC class II and costimulatory molecule B7-1/B7-2 expression [12]. Further, IL-10 suppresses IL-12 production in vitro, and in vivo studies have shown that IL-10 is a critical cytokine which protects the host from inflammation-mediated damage [13], [14]. Fernandes and Baldwin [15] using anti-IL-10 monoclonal antibodies have demonstrated that neutralization of IL-10 in vivo resulted in up to 10-fold fewer bacteria in the spleens of BALB/c mice infected with the virulent *B. abortus* strain 2308. Additionally, exogenous recombinant IL-10 inhibited the ability of peritoneal macrophages to control intracellular *Brucella*. Bacterial recognition by macrophages and dendritic cells activates intracellular signaling pathways that culminate in the induction of inflammatory cytokines, chemokines, interferons and upregulation of co-stimulatory molecules. Huang et al [16] demonstrated that IL-10 down-regulates the frequency and duration of IL-12 production by dendritic cells activated with HKBa (heat-killed *Brucella*). IL-10 production represents a potent autoregulatory feedback loop that protects against excessive inflammation and potential tissue destruction during proinflammatory Th1-driven immune responses in infections [17].

Although not life-threatening, brucellosis can cause disease with relapses of an undulant fever and lifelong complications [7]. The formation of granulomas is an important component of coordinated antibacterial defenses, in which lymphocytes cooperate with macrophages to restrain bacterial growth. Previous studies have described hepatic and splenic microgranulomas during systemic infections with pathogenic *Brucella* spp. in the mouse [18]. Therefore, the main goal of this study was to evaluate the role of endogenous IL-10 in proinflammatory cytokine production, bacterial clearance and liver pathology following *Brucella* infection. We show here that IL-10 modulates the immune response to *B. abortus* infection. Our results demonstrated that infected IL-10 KO mice maintain a reduced bacterial load in spleens which is preceded by an enhanced proinflammatory cytokine response. In contrast, at later stage of infection IL-10 KO mice showed a reduction in granuloma numbers compared to wild-type animals which coincides with increased numbers of Treg cells and TGF-*β* expression in splenocytes.

## Materials and Methods

### Ethics Statement

This study was carried out in strict accordance with the Brazilian laws 6638 and 9605 in Animal Experimentation. The protocol was approved by the Committee on the Ethics of Animal Experiments of the Federal University of Minas Gerais (Permit Number: CETEA 103/2011).

### Mice, Cell Culture and Bacteria

IL-10 KO mice from the 129Sv/Ev background were gifted by Dr. Donna Marie McCafferty (Gastroenterology Group, Calgary University, Calgary, Canada) as described previously [19]. The wild-type strain 129Sv/Ev mice were purchased from the Federal University of Minas Gerais animal facility (UFMG, Belo Horizonte, Brazil). Previously, we have demonstrated that the course of *B. abortus* infection is similar in BALB/c, C57BL/6 or 129Sv/Ev mice [20]. Genetically deficient and control mice were maintained at UFMG and used at 6–8 week of age. Bone marrow cells were obtained from femora and tibia of 129Sv/Ev or IL-10 KO mice and they were derived in dendritic cells (BMDCs) as described by Macedo et al. [21]. Brieﬂy, BM cells were cultured in DMEM (Gibco, Carlsbad, CA) containing 10% FBS (HyClone, Logan, UT), 100 U/ml penicillin, and 100 µg/ml streptomycin plus 20 ng/ml murine recombinant granulocyte-M-CSF (GM-CSF). Petri dishes containing 1×10^7^ cells were incubated at 37°C in an atmosphere of 5% CO2. At day 3 of incubation, 5 ml of fresh complete medium containing GM-CSF was added, and on days 6 and 8, 3 ml of medium was removed from the culture and replaced with fresh supplemented medium containing GM-CSF. At day 10, nonadherent cells were harvested and used to seed round-bottom 96-well culture plates (3×10^5^ cells/well) and the cells were infected with *Brucella abortus* S2308 strain (MOI 1∶100) or stimulated with 1 µg/ml of *E. coli* LPS. Culture supernatants of BMDCs were collected after 24 hours of stimulation and assayed for the concentrations of IL-10, IL-12p40 or TNF-α by ELISA (R&D Systems). *B. abortus* virulent strain 2308 was obtained from our own laboratory collection. (UFMG, Belo Horizonte, Brazil). They were grown in *Brucella* broth medium (BD-Pharmingen, San Diego, CA) for 3 days at 37°C.

### Infection and Brucella counts in Spleens

Mice were infected i.p. with 1×10^6^ CFU of *B. abortus* strain 2308. To determine residual *Brucella* CFU in the spleens of mice, five animals from each group were examined at 1, 2, 3, 6 and 14 weeks after infection. Spleens from individual animals were homogenized in PBS, 10-fold serially diluted, and plated on *Brucella* broth agar (Difco, BD-Pharmingen, San Diego, CA). Plates were incubated at 37°C and the number of CFU was counted after 3 days as previously described [9]. Only for cytokine analysis in mouse sera the animals were infected with 1×10^9^ CFU of *B. abortus* strain 2308.

### IL-10, IFN-γ, IL-17, TGF-β1 and TNF-α Production by Splenocytes

Splenocyte cultures from IL-10 KO or wild-type mice were stimulated by addition of 10^2^ live *B. abortus* strain S2308 per cell or 5 µg/ml of ConA (Sigma-Aldrich, St. Louis, MO) in a total volume of 200 µl of medium/well. Unstimulated cells were used as a negative control. Spleen cells were incubated at 37°C with 5% CO_2._ Levels of IL-10, IFN-γ, IL-17, and TNF-α in the supernatants were measured using a commercially available ELISA Duoset kit (R&D Systems, Minnesota, MN) and TGF-β1 using the BD Bioscience ELISA kit (San Jose, California, USA).

### IL-17, IFN-γ, TNF-α and IL-12 Production *in vivo*

To determine in vivo production of IL-17, IFN-γ, TNF-α and IL-12p40, blood was collected from retro-orbital plexus of IL-10 KO and wild-type mice infected i.p. with 1×10^9^ CFU of *B. abortus* strain 2308. To obtain purified serum, total blood collected in sterile tubes was maintained during 15 minutes at 37°C, 30 minutes at 4°C and then they were centrifuged at 4400 rpm during 13 minutes. The supernatant corresponding to purified serum was collected in a new sterile tube and kept at -70°C for cytokine analysis. IL-17, IFN-γ, TNF-α and IL-12p40 present in the serum were measured using the commercially available ELISA Duoset kit (R&D Systems, Minnesota, MN).

### Real-Time RT-PCR

Splenocytes from *Brucella* infected IL-10 KO and wild-type mice were snap-frozen and stored in −80°C freezer until RNA isolation. Cells were homogenized TRIzol reagent (Invitrogen) to isolate total RNA. Reverse transcription of 1µg from total RNA was performed using illustra™ Ready-To-Go RT-PCR Beads (GE Healthcare, Buckinghamshire, UK). Real-Time RT-PCR was conducted in a final volume of 10µL containing the following: SYBR® Green PCR Master Mix (Applied Biosystems, Foster City, CA), oligo-dT cDNA as the PCR template and 20µM of primers. The PCR reaction was performed with ABI 7900 Real-Time PCR System (Applied Biosystems, Foster City, CA), using the following cycling parameters: 60°C for 10 min, 95°C for 10 min, 40 cycles of 95°C for 15 sec and 60°C for 1 min, and a dissociation stage of 95°C for 15 sec, 60°C for 1 min, 95°C for 15 sec, 60°C for 15 sec. Primers were used to amplify a specific 100–120-bp fragment corresponding to specific gene targets as follows: *TGF-β1* F: 5′-TGACGTCACTGGAGTTGTACG-3′, *TGF-β1* R: 5′-GGTTCATGTCATGGATGGTGC-3′, *β-Actin* F: 5′-AGGTGTGCACCTTTTATTGGTCTCAA-3′, *β-Actin* R: 5′-TGTATGAAGGTTTGGTCTCCCT-3′. All data are presented as relative expression units after normalization to the *β-actin* gene. PCR measurements were conducted in triplicate. The differences in the relative expression of *TGF-β1* were analyzed by analysis of variance (ANOVA) followed by Tukey’s test (*P<*0.05).

### Fluorescence-activated Cell Sorter (FACS) Analysis

Infected and non-infected spleens from IL-10 KO and wild-type mice at 1, 2, 3 and 6 weeks post-infection with *B. abortus* were collected, homogenized in PBS and filtered through a 70µm cell strainer. Red blood cells were lysed with ACK buffer. The cell suspension was washed in RPMI 1640 and adjusted to 1×10^6^ cells per well for immunostaining. For CD4+CD25+Foxp3+ staining, the cells were first blocked with anti-mouse CD16/CD32 MAbs (Fc-Block) and stained for surface markers using FITC-labeled anti-mouse CD4 (clone GK 1.5, BD Bioscience) and Cy5-labeled anti-mouse CD25 (clone PC 61.5, BD Bioscience) MAbs or their isotype controls, which were incubated for 20 min at 4°C with antibody solution (PBS 0.15 M, 0.5% bovine serum albumin, 2 mM NaN3). Surface-stained cells were washed twice with 0.15 M PBS and incubated with fixation/permeabilization buffer (eBioscience) for 30 min at 4°C. Anti-Foxp3-PE-labeled antibodies in permeabilization buffer (clone FJK-16s, eBioscience) were added to cells and then incubated for 30 min at 4°C. Cells were washed twice with 150 µl of antibody dilution buffer (eBioscience) and ressuspended in 150µl of PBS. To determine the cell source of IL-10, IL-17 and IFN-γ, at one-week post-infection, a similar protocol was performed staining for surface markers using fluorescein isothiocyanate (FITC)-conjugated anti-mouse CD4 (clone GK1.5; eBioscience, San Diego, CA), biotin-conjugated CD8a (clone 53-6.7; eBioscience, San Diego, CA), (FITC)-conjugated anti-mouse CD19 (clone MB19-1; eBioscience, San Diego, CA), (FITC)-conjugated anti-mouse CD11c (clone N418; eBioscience, San Diego, CA), (FITC)-conjugated anti-mouse CD11b (clone M1/70; eBioscience, San Diego, CA) or biotin-conjugated F4/80 (clone BM8; eBioscience, San Diego, CA). After 20 min, cells were washed, fixed using a 4% formaldehyde solution, and permeabilized with a 0.5% saponin solution in PBS. Cells were stained with phycoerythrin (PE)-conjugated anti-mouse IFN-γ (clone XMG1.2; BD Pharmingen, San Diego, CA), PE-conjugated anti-mouse IL-10 (clone JES5-16E3; BD Pharmingen, San Diego, CA) or PE-conjugated anti-mouse IL-17F (clone eBio18F10; eBioscience, San Diego, CA). Data acquisition (30,000 events) was performed using FACScan ﬂow cytometer (Becton Dickinson, San Jose, CA) and data were analyzed using the FlowJo Software (Tree Star, Ashland, OR).

### Histopathology and Immunohistochemistry Assays

The medial lobes of the *B. abortus*-infected mice liver were collected at 1, 2, 3 and 6 weeks postinfection, fixed in 10% buffered formaldehyde solution, dehydrated, diaphanized, and embedded in paraffin. Four-micrometer-thick tissue sections were stained with hematoxylin and eosin (H&E). The total number of granulomas present in histological liver sections was determined using an Axiolab microscope (Carl Zeiss, Oberkochen, Germany) with a 40×objective lens. Digital images of the sides were captured at a resolution of 300 dots/inches using a scanner (HP Scanjet 2400; Hewlett-Packard, Love-land, CO). For histopathological evaluation, the slides were examined by using blinded sample reading. Volumetric proportion of liver tissue components, including portal space, central lobular vein, granuloma, necrosis and parenchyma were measured with a 25-point circular grid (Zeiss KPL ocular 6.3X with 25 points) over 10 randomly selected microscopic fields (20×objective) for a total of 250 points per slide. Immunohistochemistry was performed as previously described [22]. Briefly, liver sections were hydrated and incubated with 10% hydrogen peroxide in PBS for 30 min. After being washed with PBS, slides were transferred to a humid chamber at room temperature, incubated with 25 mg/ml of skim milk for 45 min, and then incubated with a primary antibody for 30 min. For immunolabeling, diluted (1∶5,000) serum from a rabbit experimentally inoculated with *B. abortus* S19 strain was used as polyclonal anti-*B. abortus* antibody. Then, tissue sections were washed with PBS, incubated with secondary antibody for 20 min, washed again with PBS, and incubated for 20 min with streptavidin-peroxidase from a commercial kit (LSAB^+^ kit; Dako Corporation, Carpinteria, CA). The reaction was revealed using 0.024% diaminobenzidine (DAB; Sigma), and sections were counterstained with Mayer’s hematoxylin.

### Statistical Analysis

Statistically significant differences between the results obtained with IL-10 KO mice and wild-type animals were evaluated by ANOVA followed by Tukey’s *post hoc* test (p<0.05). For histomorphometry, volumetric proportion data expressed as percentage were submitted to analysis of variance after angular transformation and comparison by ANOVA followed by Tukey’s *post hoc* test (p<0.05).

## Results

### *Brucella abortus* Induces IL-10 Production in Dendritic Cells and Splenocytes

**Figure 1:**
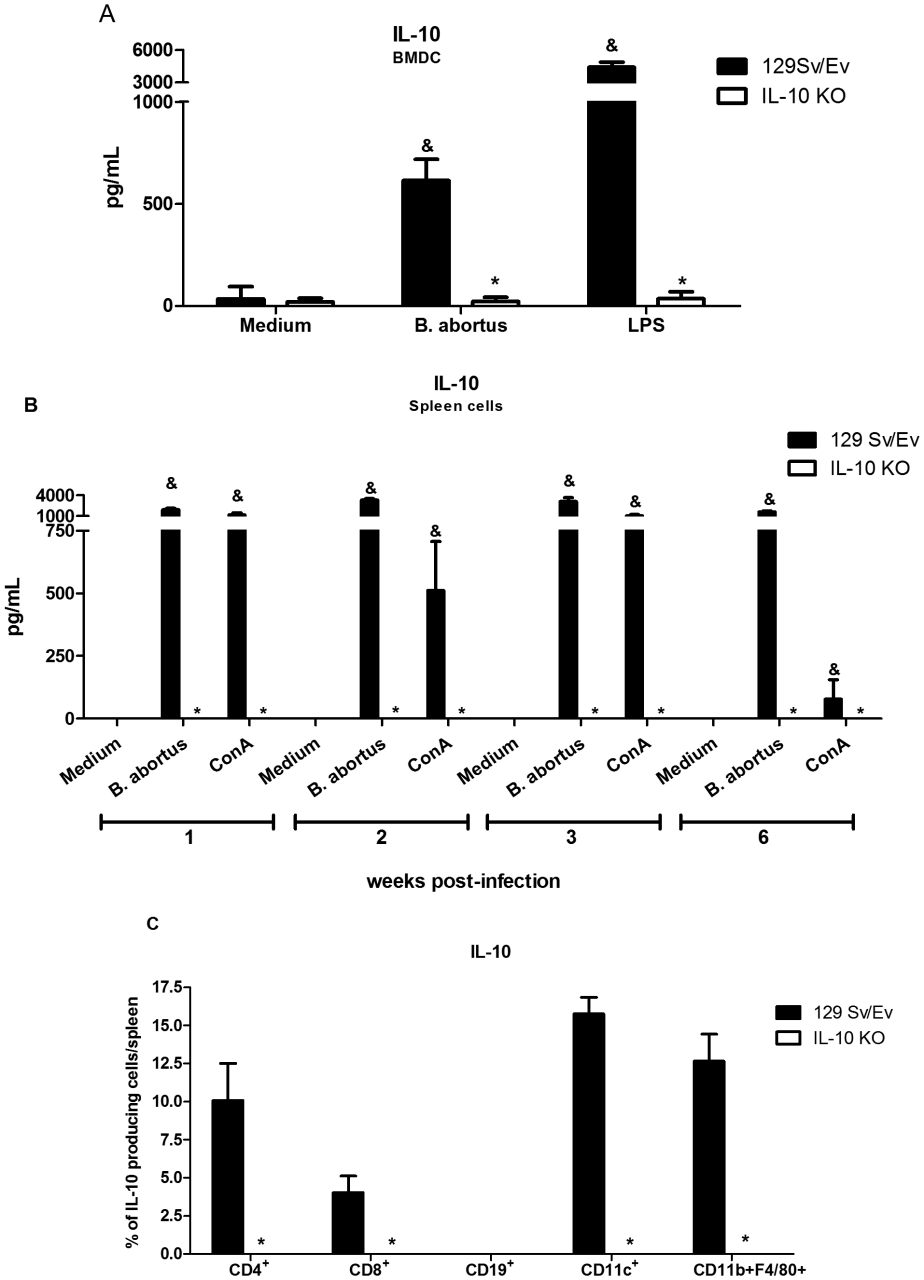
*Brucella* induces IL-10 production in BMDCs and spleen cells in 129 Sv/Ev mice. (A) Bone marrow cells from wild-type or IL-10 KO mice were differentiated in dendritic cells (BMDC) and infected with *B. abortus* (MOI 1∶100) or stimulated with *E. coli* LPS (1 µg/ml). IL-10 was measured by ELISA at 24 hours after antigen stimulation. (B) Spleen cells from *B. abortus* infected mice at 1, 2, 3 or 6 weeks postinfection were cultured with 10^2^ bacteria/cell, ConA (5 µg/ml) or medium alone for 72 hours. Supernatants were harvested for measuring IL-10 by ELISA. The level of IL-10 at week 0 was below 30 pg/ml. Error bars represent the mean ±SD. Similar results were obtained in four-independent experiments. Statistically significant differences of IL-10 levels from IL-10 KO mice compared to wild-type are denoted by an asterisk (p<0.05). Differences of IL-10 levels from wild-type mice stimulated with *Brucella* or the positive control compared to medium alone are denoted by & (p<0.05). (C) Flow cytometry analysis of CD4+, CD8+, CD19+, CD11c+ and CD11b+/F4/80+ cells producing IL-10 was carried out in splenocytes from *Brucella*-infected mice at one-week post-infection. Statistically significant differences of IL-10 levels from IL-10 KO mice compared to wild-type are denoted by an asterisk (p<0.05).

To investigate whether *B. abortus* induces IL-10, BMDC (bone marrow-derived dendritic cells) or splenocytes were exposed to bacteria and IL-10 was measured. Wild-type (129 Sv/Ev) BMDC as well as splenocytes infected with *B. abortus* strain 2308 displayed increased production of IL-10 as measured by ELISA. In infected BMDC, IL-10 was observed at 24 hours post-infection and in splenocytes kinetics of cytokine secretion was measured at 1, 2, 3 and 6 weeks postinfection. Both cell types produce great amounts of IL-10 after *Brucella* infection (Figure 1A and 1B). To identify the cell source producing IL-10 in splenocyte population we performed flow cytometry. FACS analysis revealed that CD4+ T cells, macrophages and dendritic cells are the major IL-10 producers within splenocytes (Figure 1C). Our results corroborate with previous studies that showed that HKBA (heat-killed *Brucella abortus*) induced IL-10 mRNA in vivo and IL-10 protein in vitro [23], [24]. As expected *B. abortus* induced no IL-10 production on BMDC and splenocytes cells of IL-10 KO mice. These findings demonstrate that in vivo and in vitro live *B. abortus* induces IL-10 production in immune cells.

### Elevated Proinflammatory Cytokine Production in IL-10 KO Dendritic Cells

**Figure 2:**
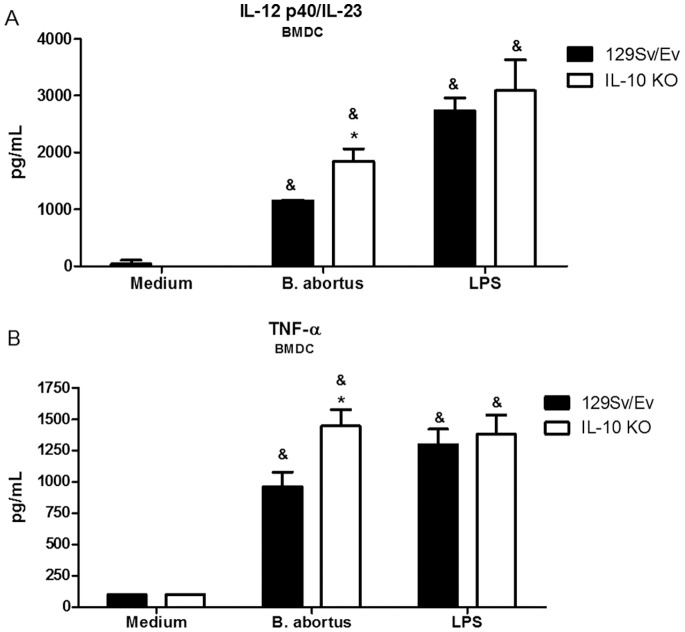
IL-10 is required to control proinflammatory cytokine production by BMDCs infected with *B. abortus*. Bone marrow cells from wild-type or IL-10 KO mice were differentiated in dendritic cells and infected with *B. abortus* (MOI, 1∶100) or stimulated with *E. coli* LPS (1 µg/ml). Supernatants were harvested for measuring IL-12 (A) or TNF-α (B) at 24 hours after infection/stimulation by ELISA. Error bars represent the mean ±SD. Similar results were obtained in four-independent experiments. Statistically significant differences relative to medium (nonstimulated cells) are denoted by & (p<0.05) and differences from IL-10 KO mice compared to wild-type are indicated by an asterisk (p<0.05).

The recognition of *Brucella* by innate immune cells, such as macrophages and dendritic cells, results in activation and the concomitant production of proinflammatory cytokines [21]. In order to evaluate the role of endogenous IL-10 in proinflammatory cytokine production, BMDC of wild-type and IL-10 KO mice were stimulated with live *B. abortus*. As shown in Figure 2, lack of IL-10 resulted in augmented secretion of IL-12 and TNF-α by IL-10 KO BMDC when compared to cells of wild-type mice. In conclusion, in the absence of IL-10, IL-12 and TNF-α production was enhanced by BMDC.

### The Absence of IL-10 Enhances Host Protection to *Brucella abortus* Infection *in vivo*

**Figure 3:**
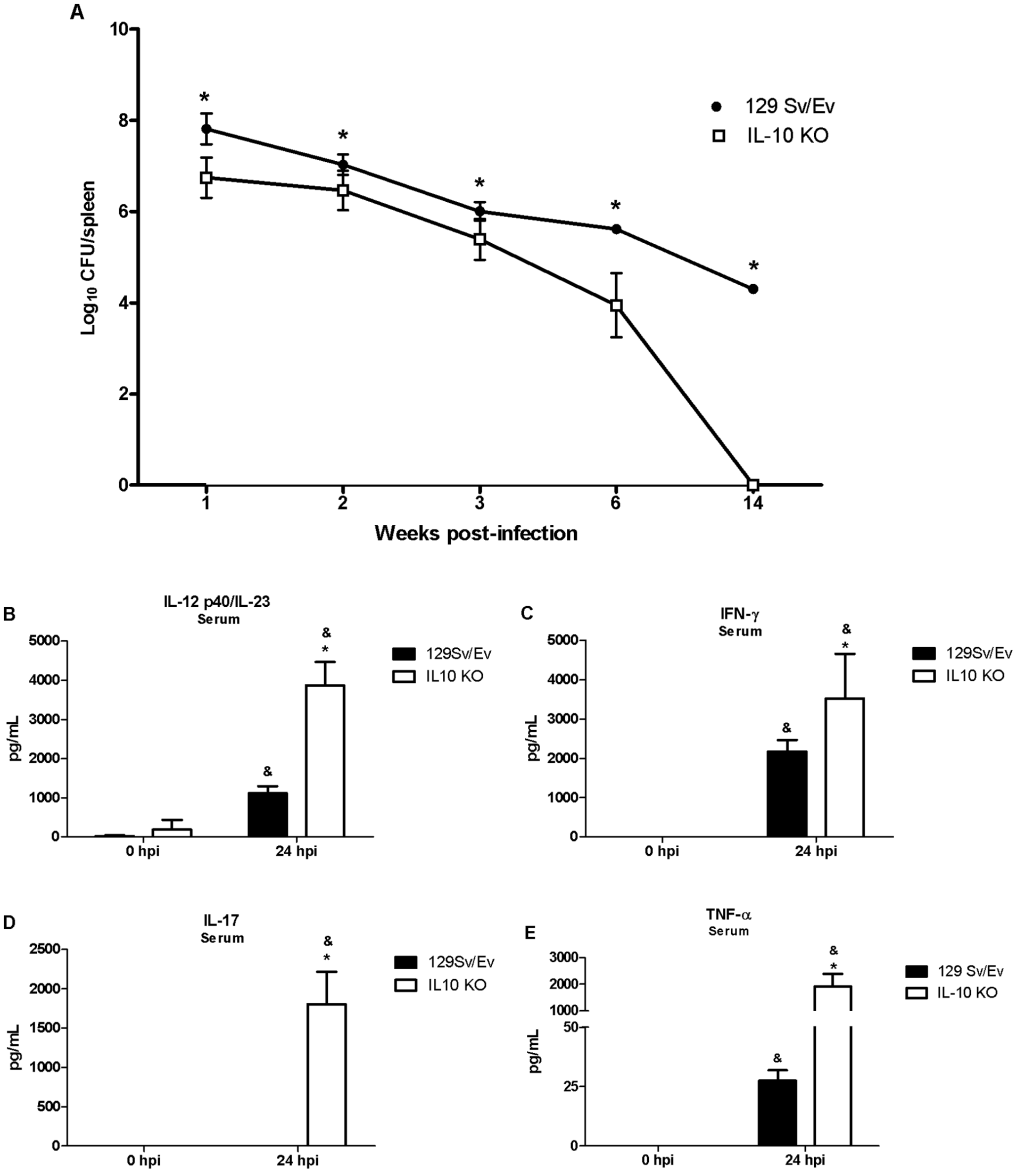
Bacterial burden and IL-12, TNF-α, IFN-γ and IL-17 production in *Brucella*-infected IL-10 KO mice. Five wild-type or IL-10 KO mice were infected i.p. with a dose of 10^6^ CFU of *B. abortus* for bacterial burden analysis or a dose of 10^9^ CFU of *B. abortus* for serum cytokine analysis. (A) Spleens were harvested at 1, 2, 3, 6 or 14 weeks post-infection, and the number of CFU in disrupted tissue was determined by 10-fold serial dilution and plating. Statistically significant differences in CFU of IL-10 KO compared to wild-type mice are denoted by an asterisk (p<0.05). IL-12 (B), IFN-γ (C), IL-17 (D) or TNF-α (E) production in mice sera were measured by ELISA at 24 hours postinfection. Error bars represent the mean ±SD. Similar results were obtained in four-independent experiments. Statistically significant differences of cytokine levels from IL-10 KO mice compared to wild-type are denoted by an asterisk (p<0.05) and statistically significant differences to time 0 hour are denoted by & (p<0.05).

To study the role of IL-10 in host response in vivo against *Brucella* infection, we infected IL-10 KO and wild-type mice with 1×10^6^ *B. abortus* strain 2308. Comparison of colony-forming units (cfu) was analyzed from spleens of IL-10 KO and wild-type mice at 1, 2, 3, 6 and 14 weeks postinfection. Figure 3A shows reduced bacterial numbers at all time intervals studied in IL-10 KO compared to wild-type mice. However, bacterial load recovery was 1.67 and 4.29 logs lower at 6 and 14 weeks, respectively, in IL-10 KO mice compared to wild-type animals. As a matter of fact at 14 weeks postinfection, we were not able to detect *Brucella* CFU in spleens of IL-10 KO mice. These results demonstrated that IL-10 has a detrimental effect on host control of *B. abortus* infection in mice.

Protective immunity against infection by *B. abortus* is directly related to the induction of a type 1 pattern of immune response [9], [11]. Thus, to evaluate the role of IL-10 in regulating a type 1 immune response during *B. abortus* infection, the production of IL-12, IL-17, TNF-α and IFN-γ *in vivo* was assessed. IL-10 KO and wild-type mice were infected with *B. abortus* and 24 hours after infection serum IL-12, IL-17, TNF-α and IFN-γ levels were determined in these mice. IL-12, IL-17, TNF-α and IFN-γ production in IL-10 KO mice was greatly augmented compared to wild-type animals (Figure 3B–E). This result demonstrates that lack of IL-10 results in enhanced IL-12, IL-17, TNF-α and IFN-γ synthesis during *Brucella* infection in vivo.

### Lack of Endogenous IL-10 Leads to Increased IFN-γ, TNF-α and IL-17 Production by Spleen Cells

**Figure 4:**
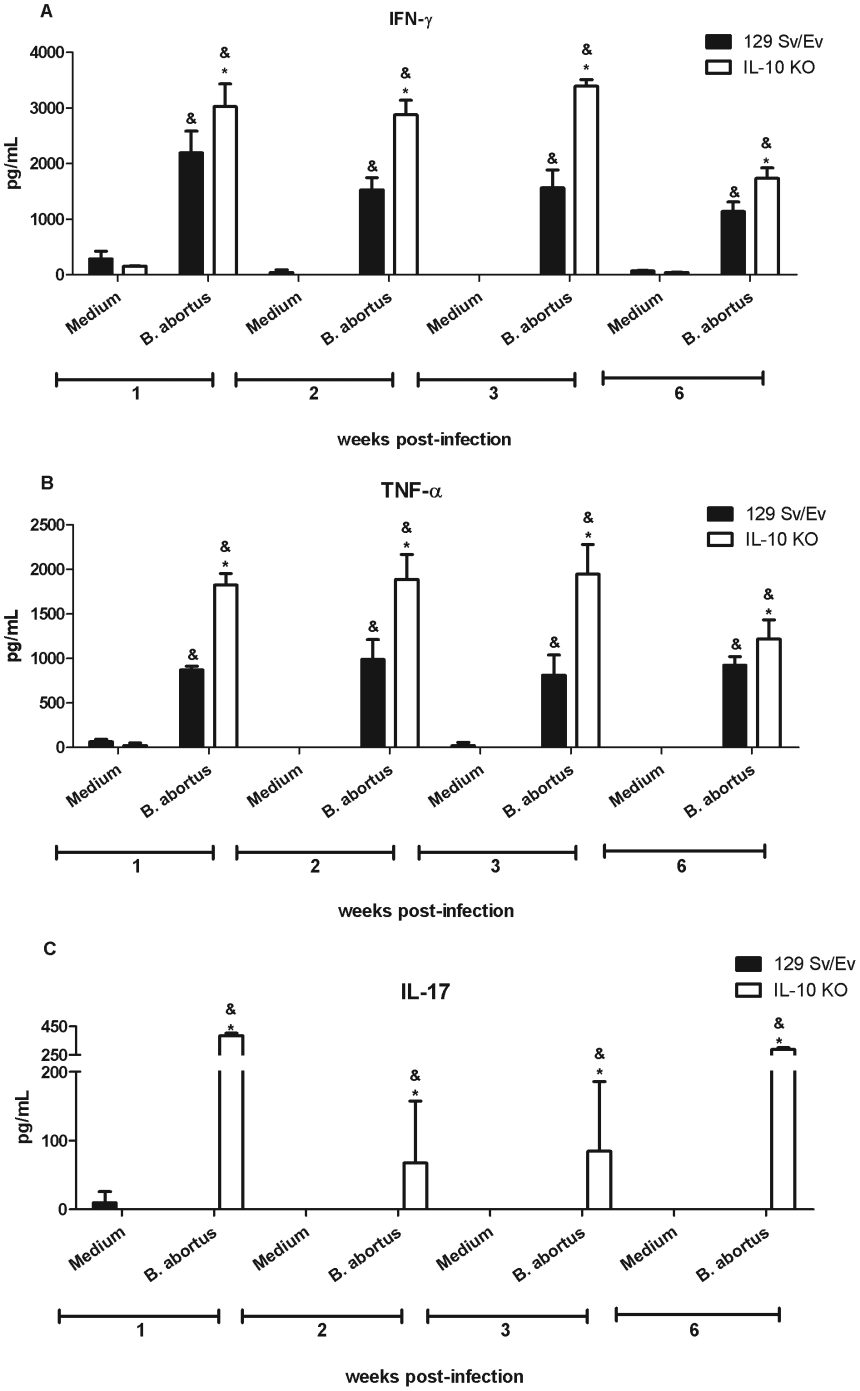
Endogenous IL-10 is required to control proinflammatory cytokine production by splenocytes infected with *B. abortus*. Spleen cells from wild-type or IL-10 KO *Brucella*-infected mice at 1, 2, 3 or 6 weeks postinfection were stimulated with *B. abortus* (MOI 1∶100) or medium alone as a negative control. Splenocyte supernatants were harvested for measuring IFN-γ (A), TNF-α (B) or IL-17 (C) levels by ELISA. Data are expressed the mean ±SD for five animals per group. The level of IFN-γ and TNF-α at week 0 was below 30 pg/ml and for IL-17 was undetectable. These results are representative of four independent experiments. Statistically significant differences relative to medium alone are denoted by & and differences of IL-10 KO compared to wild-type mice are indicated by an asterisk (p*<*0.05).

The production of proinflammatory cytokines such as IFN-γ and TNF-α, is associated with control of *Brucella abortus* infection [25], [26]. Herein, we showed that IL-10 KO mice were more resistant to *B. abortus* infection than the wild-type animals. To determine whether this resistance is related to enhanced proinflammatory cytokine production during recall responses in vitro, splenocytes from *Brucella*-primed IL-10 KO and wild-type animals were infected and IFN-γ, TNF-α, IL-17 was measured in cell supernatants. IL-10 KO cells produced higher levels of TNF-α, IFN-γ and IL-17 when stimulated with *B. abortus* at 1-, 2-, 3- and 6- weeks postinfection compared to wild-type cells (Fig. 4A, B and C). Additionally, we demonstrated that CD4+ T cells are the major source of IFN-γ and IL-17 within the lymphocyte population in the spleen (Table 1). Together, these results confirm the ability of IL-10 to act directly or indirectly reducing the production of IFN-γ, TNF-α and IL-17 during *B. abortus* infection [16].

**Table 1:** Mean percentages ± SD of CD4^+^, CD8^+^ or CD19^+^ cells producing IFN-γ or IL-17 in 129 Sv/Ev or IL-10 KO splenocytes at one week post-infection with *Brucella abortus*.

	IFN-γ		IL-17	
Cell population	129 Sv/Ev	IL-10 KO	129 Sv/Ev	IL-10 KO
CD4^+^	10.51±5.24	33.03±6.54^a^	3.37±0.58	13.13±2.65^a^
CD8^+^	3.96±1.57	12.36±2.10^a^	3.53±0.89	11.36±0.95^a^
CD19^+^	0.43±0.04	1.52±0.96	0±0	0.58±0.11

a Statistically significant compared to 129 Sv/Ev mice.

### IL-10 KO mice show a Reduction in Liver Granuloma Numbers at Later Stage of Infection

**Figure 5:**
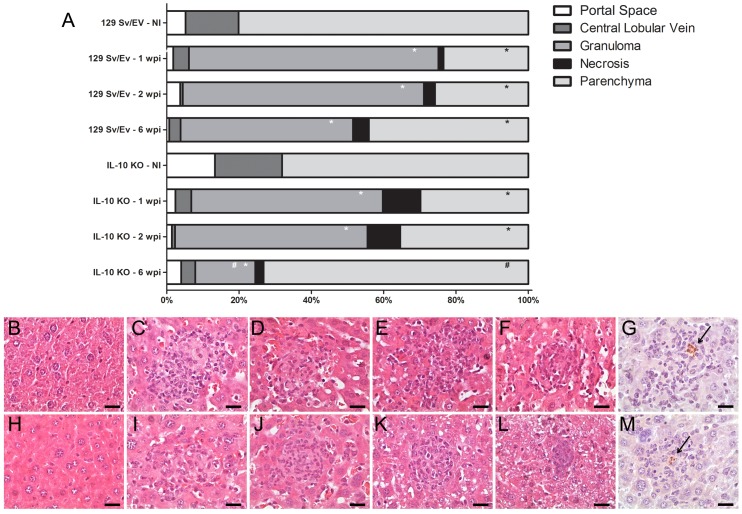
Morphometric analysis, histopathology and immunohistochemistry of hepatic tissue of *B. abortus* infected IL-10 KO mice. (A) Columns indicate volumetric proportions of tissue components. The number of portal space, central lobular vein, granuloma, necrosis and parenchyma were evaluated and transformed in percentage at 1, 2 or 6 weeks postinfection. Statistically significant differences relative to non-infected group (NI) are represented by an asterisk (p*<*0.05). Differences relative to granuloma number from IL-10 KO mice compared to wild-type mice at six-week postinfection are indicated by #. Similar results were obtained in two-independent experiments. (B-F) Representative of hematoxylin- and-eosin-stained sections of hepatic tissue from wild-type mice uninfected (B) or infected at one- (C), two- (D), three- (E) or six-weeks (F). (H-L) Representative of hematoxylin- and eosin-stained sections of hepatic tissue from IL-10 KO mice uninfected (H) or infected at one- (I), two- (J), three- (K) or six-weeks (L). Immunohistochemistry sections of hepatic tissue from wild-type (G) and IL-10 KO (M) mice containing the *B. abortus* inside the granuloma. The arrows indicate the *B. abortus* within the granuloma. Scale bars: 20 µm.

*B. abortus* infection is associated with the formation of focal granulomatous lesions in the spleen, liver, and lymphoid tissues of both humans and rodents, starting at 2 to 3 weeks postinfection [18]. To determine whether IL-10 can alter liver pathology during *B. abortus* infection, we performed histopathologic analysis of liver tissue from IL-10 KO and wild-type mice at 1, 2, 3 and 6 weeks postinfection. We evaluated the percentage of portal space, central lobular vein, granuloma, necrosis and parenchyma in the livers tissue sections of animals infected with *B. abortus* and uninfected. Infection with *B. abortus* resulted in the formation of liver granulomas and necrotic lesions in both IL-10 KO and wild-type mice which was associated with a decrease in volumetric proportion of the parenchyma (Figure 5A). We did not observe a significant difference in granuloma formation in IL-10 KO mice when compared to wild-type animals at 1- and 2-weeks postinfection. However, at six-week postinfection we detected a reduction in granuloma numbers in IL-10 KO compared to wild-type animals and an increase in the in volumetric proportion of the parenchyma. Histopathological lesions during *Brucella* infection usually are associated with bacterial load. To determine the relation between granuloma formation and the bacteria present in the granuloma we performed immunohistochemistry to immunolabel *B. abortus*. Figures 5G and M showed the presence of *B. abortus* in the granulomatous lesions presented in liver of IL-10 KO and wild-type mice. The detection of intralesional bacteria confirms that the inflammatory lesions described in this study are due to systemic *B. abortus* infection.

### IL-10 Deficiency Enhances CD4+CD25+foxp3+ T cell Numbers and Expression of TGF-β1 Following *Brucella* Infection

**Figure 6:**
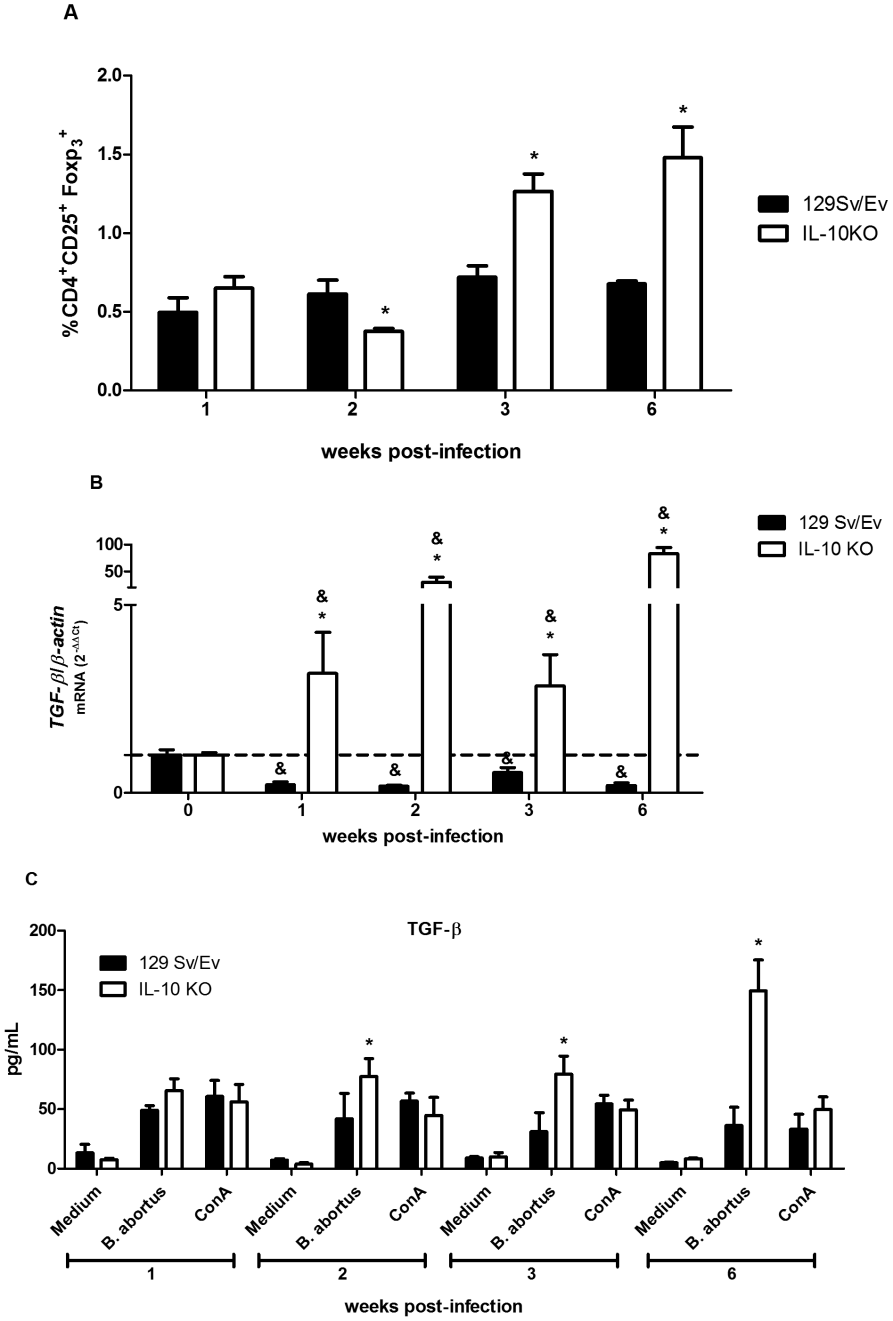
Increased percentage of CD4^+^CD25^+^Foxp3^+^ T cells and *TGF-β* expression in IL-10 KO mice infected with *B. abortus*. (A) Flow cytometry analysis of CD4^+^CD25^+^Foxp3^+^ T cells was carried out in splenocytes from infected (I) or non-infected (NI) mice at 1, 2, 3 or 6 weeks post-infection. Results are expressed as arbitrary units from relative percentage of I/NI CD4^+^CD25^+^Foxp3^+^ T cells encountered in the spleens at indicated time points. The ratio between the percentage of CD4^+^CD25^+^Foxp3^+^ T cells/spleen in I/NI animals was used because of the variation observed on the percentage of these cells in NI mice at different time intervals tested due to the age difference during the duration of the experiment. The experiments were conduct in triplicate and three independent experiments were performed with similar results. Error bars represent the mean ±SD. Statistically significant differences between wild-type and IL-10 KO mice are denoted by an asterisk (p<0.05). (B) Splenocytes from *B. abortus* infected mice at 1, 2, 3 or 6 weeks postinfection were isolated and total RNA was harvested and mRNA levels of *TGF-β1* were determined by real-time RT-PCR and normalized to *β-actin*. Error bars represent the mean ±SD. Similar results were obtained in three-independent experiments. Statistically significant differences of *TGF-β1* expression from IL-10 KO mice compared to wild-type are denoted by an asterisk (p<0.05) and differences of *TGF-β* expression from infected mice compared to uninfected control (0 week) are denoted by & (p<0.05). (C) splenocyte culture supernatants were harvested for measuring TGF-β1 levels by ELISA. Data are expressed the mean ±SD for five animals per group. Error bars represent the mean ±SD. Statistically significant differences between wild-type and IL-10 KO mice are denoted by an asterisk (p<0.05).

The CD4^+^ CD25^+^ Foxp3^+^ T cells termed regulatory T cells (Treg) have the role of suppressor activity in maintaining tolerance to self molecules of the host, and in maintaining homeostasis of the immune system [27], [28]. Natural Treg cells possess the suppressive effect mediated primarily by cell-to-cell contact, but also through the involvement of cytokines in the activation of this suppressive effect [29]. However, induced regulatory T cells are related to host immunity after an infection or alteration of homeostasis and/or tolerance to microbes or immunostimulating molecules. Herein, we performed FACS analysis to demonstrate the percentage of Treg cells (infected/non-infected mice) present in spleens from IL-10 KO mice compared to wild-type. Our results demonstrated an increased in Treg cell population in IL-10 KO animals compared to wild-type at 3 and 6 weeks post-infection (Figure 6A). Like IL-10, TGF-β is considered as a regulatory cytokine [30]. To assess the role of endogenous IL-10 in *TGF-β1* expression and secretion during *Brucella abortus* infection, we performed real-time PCR and ELISA in spleen cells from infected mice at 1, 2, 3 and 6 weeks post-infection. Expression analysis demonstrated that *TGF-β1* transcripts were increased in IL-10 KO mice during all time intervals postinfection compared to wild-type mice (Figure 6B). Regarding TGF-β1 secretion, we observed augmented production of this cytokine in IL-10 KO spleen cells at 2, 3, and 6 weeks post-infection compared to wild-type animals (Figure 6C). Together, these results suggest that in absence of IL-10 other regulatory components of the immune system such as Treg cells and TGF-β are activated during *Brucella* infection.

## Discussion

IL-10 has emerged as a key immunoregulator ameliorating the excessive Th1 responses that are responsible for much of the immunpathology during several infections [2]. Previous reports have shown that either heat-killed *Brucella* or live bacteria was able to induce IL-10 synthesis in CD4+ T cells, splenocytes and B cells [15],[23],[31]. In this study, we determined the time-course of IL-10 synthesis during *B. abortus* infection. Wild-type mice were infected with virulent strain S2308 and IL-10 production was measured at 1, 2, 3 and 6-weeks postinfection. High levels of IL-10 were detected in all time intervals studied and no IL-10 was observed in IL-10 KO spleen cells as expected. Furthermore, FACS analysis demonstrated that CD4+ T cells, macrophages and dendritic cells are the major sources of IL-10 within splenocytes. Additionally, we provided evidence that BMDC from wild-type mice produced elevated levels of IL-10 determining that another cell type could take part in IL-10 production during *Brucella* infection.

By acting on DCs and macrophages, IL-10 inhibits the development of Th1 and Th2 type of immune responses [32]. Because Th1 responses are critical to control *Brucella* infection, we undertook a detailed examination of IL-12 and TNF-α production in BMDC and IFN-γ, TNF-α and IL-17 production in spleen cells. Twenty hours after *Brucella* infection, enhanced IL-12 and TNF-α production were detected in BMDC from IL-10 KO compared to wild-type cells. At the same time-point, elevated levels of IFN-γ, TNF-α, IL-12 and IL-17 were detected in vivo in sera of IL-10 KO compared to wild-type mice. Additionally, spleen cells from infected IL-10 KO mice secreted much higher levels of IFN-γ, TNF-α and IL-17 compared to wild-type animals. Further, FACS analysis demonstrated that CD4+ T cells are the major cell source of IFN-γ and IL-17. These results demonstrate that lack of IL-10 enhances proinflammatory cytokine production in *Brucella* infected animals. This enhanced proinflammatory response observed in absence of IL-10 was accompanied by increased resistance to *B. abortus* infection with significantly reduced bacterial load in spleens of IL-10 KO mice over the course of infection studied. As a matter of fact, at 14-weeks postinfection we could not detect *Brucella* CFU in IL-10 KO mouse spleens. Our findings provide evidence that IL-10 has a detrimental effect on control of *Brucella* in vivo. By administering anti-IL-10 MAb in vivo Fernandes & Baldwin [15] have demonstrated similar results at one-week postinfection. Regarding IL-17, previous reports have implicated this cytokine in host protection against *Brucella* infection in mouse and humans [33], [34]. In our study, it is clear that IL-17 is tightly regulated by IL-10 since we do not detect this cytokine in serum or spleen cells of wild-type mice but only in IL-10 KO animals. In addition to macrophages, dendritic cells and B cells, IL-10 can be produced by Th1, Th3 and Th17 cells. However, the fact that Th1, Th2 and Th17 cells are dependent on DC- and macrophage-derived factors that are down-regulated by IL-10, it is indicative of a negative feedback loop that ensures that effector T cell responses do not result in immunopathology [32].

Pathological manifestation characteristic of *Brucella* infection is granulomatous inflammation associated with bacterial load [18]. Histological changes in the liver are characterized by the development of small granulomas and an influx of polymorphonuclear leukocytes (PMN) and monocytes. In this study, we observed in livers of IL-10 KO and wild-type mice an acute inflammatory response characterized by increased in granuloma numbers and necrosis at 1- and 2-weeks postinfection when compared to uninfected mice. Additionally, at these intervals of infection we observed a slight increase in necrotic tissues in IL-10 KO compared to wild-type livers which was not statistically significant. However, at later stage of infection (six-weeks), we detected a significant reduction in granuloma numbers in IL-10 KO compared to wild-type livers. This reduction in liver pathology in IL-10 KO mice was accompanied by an increased numbers of CD4+CD25+foxp3+ T cells and an enhanced expression and secretion of TGF-β1 in splenocytes. Recently, Xavier et al [35] have demonstrated a critical role for IL-10 in modulating pathology during early *B. abortus* infection. Although the absence of IL-10 leads to a better clearance of some pathogens with no enhanced immunopathology [36], during other infections lack of IL-10 can be accompanied by an immunopathology that is detrimental to the host but does not necessarily affect the pathogen load [3]. Here, we hypothesize that reduction on liver pathology in IL-10 KO mice could be explained in part by other regulatory components of the immune system such as Treg cells and/or TGF-β1 production or even by the reduction of bacterial load during the course of infection.

In conclusion, we showed here that IL-10 regulates the immune response to *B. abortus* infection. This cytokine modulates proinflammatory cytokine production and in the absence of IL-10, mice reduce bacterial load leading to *Brucella* clearance. Lastly, at later stage of infection lack of IL-10 leads to increased number of Treg cells and TGF-β1 production which coincides with reduction in liver pathology. Future studies from our group will dissect the role of Treg cells and TGF-β in controlling host pathology during *Brucella* infection.
